# Endoscopically Based Endonasal and Transnasal Lasersurgery

**DOI:** 10.1155/DTE.7.109

**Published:** 2001

**Authors:** Hans Scherer, Juergen U. G. Hopf, Marietta Hopf

**Affiliations:** Department of Otorhinolaryngology Head and Neck Surgery University Medical Center Benjamin Franklin Freie Universitaet Berlin, Hindenburgdamm 30 Berlin 12200 Germany

## Abstract

The endoscopically based endonasal and transnasal laser surgery is a surgical procedure, which offers the ENT-specialist a safe and effective method to cure or to improve a number of diseases of the upper and middle airways. Coagulative lasers are used in contact and noncontact mode. Their light is mainly absorbed by hemoglobin but rarely by water. The laser–tissue interaction is performed via flexible glass fibers. For the delivery of the laser beam we use specially designed applicator sheaths, which incorporate the endoscope, the laser fiber and the suction channel. The procedure is controlled online via the endoscopic image on the monitor (“video-endoscopy”). The patient suffers less trauma using this treatment compared to the standard endoscopic surgery and the procedure is much quicker. Pre- and post-operative rhinomanometric and rhinoresistometric measurements reveal that the air flow rate of the nose can be improved effectively.


*Diagnostic and Therapeutic Endoscopy*, Vol. 7, pp. 109–127
Reprints available directly from the publisher
Photocopying permitted by license only

© 2001 OPA (Overseas Publishers Association) N.V.
Published by license under
the Harwood Academic Publishers imprint,
part of Gordon and Breach Publishing
a member of the Taylor & Francis Group.
All rights reserved.

### Endoscopically Based Endonasal and Transnasal Lasersurgery

HANS SCHERER*, JUERGEN U.G. HOPF and MARIETTA HOPF

*Department of Otorhinolaryngology, Head and Neck Surgery, University Medical Center Benjamin Franklin, Freie Universitaet Berlin, Hindenburgdamm 30, 12200 Berlin, Germany*

*(Received 3 October 2001; In final form 8 May 2002)*

The endoscopically based endonasal and transnasal laser surgery is a surgical procedure, which offers the ENT-specialist a safe and effective method to cure or to improve a number of diseases of the upper and middle airways. Coagulative lasers are used in contact and non-contact mode. Their light is mainly absorbed by hemoglobin but rarely by water. The laser–tissue interaction is performed via flexible glass fibers. For the delivery of the laser beam we use specially designed applicator sheaths, which incorporate the endoscope, the laser fiber and the suction channel. The procedure is controlled online via the endoscopic image on the monitor ("video-endoscopy"). The patient suffers less trauma using this treatment compared to the standard endoscopic surgery and the procedure is much quicker. Pre- and post-operative rhinomanometric and rhinoresistometric measurements reveal that the air flow rate of the nose can be improved effectively.

*Keywords*: Chronic sinusitis; Endonasal surgery; Laser surgery; Nasal turbinate; Osler's disease; Septum nasi

### INTRODUCTION

Diseases of the nose and the paranasal sinuses are common all over the world. Most patients suffer from air-way blockage (anatomical abnormalities or mucosal), hypersecretion, hypo- or anosmia, an acute, recurrent or chronic sinusitis. As disturbances of the nasal function not only affect the quality of life but also the functions of the middle and lower airway structures such as pharynx, larynx, tracheal and bronchial system, ENT-specialists are often asked to take therapeutic action.

*Corresponding author. Tel.: +49-30-8445-2431. Fax: +49-30-8445-4460.

Some problems can be cured with drugs, but surgical interventions are often needed in cases of:

- septal deviations, ridges and spurs,
- turbinal hyperplasia in environmental problems and in chronic mucosal hyperreactivity
- acute sinusitis with complications
- recurrent sinusitis
- chronic sinusitis with local or general mucosal hyperplasia and polyps
- mucoceles
- epistaxis, i.e. in Osler's disease

In the past two decades endoscopic or microscopic endonasal sinus surgery has replaced the radical forms of intervention and has become the "gold standard". Messerklinger [1–3], Wigand [4,5], Stammberger [6], Kennedy [7], Rudert [8] and others are the main contributors to the progress of this method. Through their work we have learnt more about the causes of chronic sinusitis. The micro-surgical procedure has led to a marked reduction in surgical trauma.

An additional reduction in surgical trauma could be achieved by the use of lasers in combination with rigid or flexible endoscopes [9–27], a method which will be described in this paper.

With special lasers the surgeon is able to avoid bleeding almost totally without loss of surgical options. He can work precisely and in very small areas using only a superficial anesthesia. There are cavernous bodies full of blood vessels in the three turbinates of each side of the nose and in parts of the septum. These vessels can be partly closed very effectively with the use of laser light, which is absorbed by hemoglobin. These lasers are also best for the closure of superficial vessels of the anterior nose or of branches of the sphenopalatine artery at the posterior areas of the nose in recurrent epistaxis or in Osler's disease. Lasers allow to perform surgical interventions even in patients with genetically fixed or drug-related hemophilia.

In some cases the nose is used as a transfer organ to naso-, oro- hypopharyngeal or laryngeal pathologies, especially cysts, papillomas or edema formations after irradiation. Rigid or flexible endoscopes carry the glass fibers for the laser beam.

This article describes the surgical procedure, a selection of suitable lasers together with the potential risks involved in this treatment. Furthermore, we will give the outlook for the future of endo- and transnasal laser surgery.

### MATERIALS AND METHODS

### Lasers Used in Endo- and Transnasal Surgery

A wide variety of different lasers are applicable as surgical tools, but not all of them are suitable for the endo- and transnasal surgery.

We use lasers with wavelengths below 1200 nm that show a very low absorption of photons by water (Fig. 1A). The laser light, therefore, passes through water, is scattered and delivers its energy relatively deep into the tissue. When the laser light has a wavelength in which there is a high or medium absorption by hemoglobin (450–600 nm; 800–950 nm), then the laser energy is concentrated in blood-vessels, arteries or veins, depending on the degree of absorption in oxygenated or desoxygenated blood (Fig. 1B).

For this purpose the best lasers are *Diode lasers* (810–980 nm) (medial absorption by oxy- and desoxygenated hemoglobin), the *Nd:YAG laser* (1064 nm) (no absorption in desoxygenated hemoglobin) and *Argon laser* (488/514 nm) as well as *KTP-laser* (532 nm) (high absorption in oxy- and desoxygenized hemoglobin).

The light can be delivered in a non-contact mode. In this case, the surface of the tissue is less damaged, but the effect of irradiation is inside the tissue and cannot be controlled so well. This type of laser surgery should be restricted to experienced surgeons. It is the preferred treatment method for Osler's disease, in which one should not touch the tissue because, otherwise, bleeding will occur immediately.

In the nose, usually a very high penetration depth is not desirable. In this case, we work in the contact mode. The tissue is touched with the fiber, the tip of which has been pre-blackened with

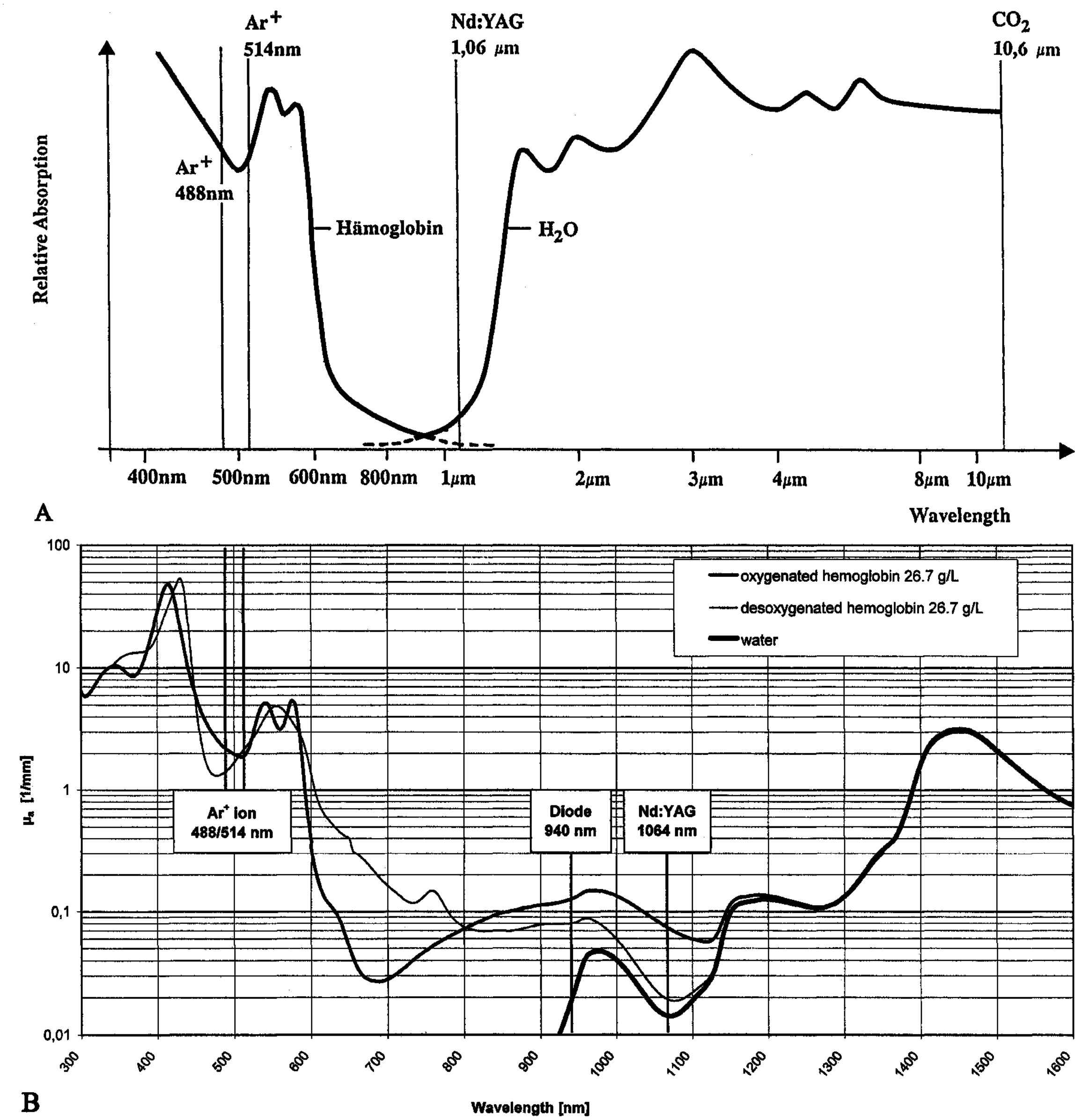

FIGURE 1 (A) Relative photon absorption in water and hemoglobin. Wavelengths of Argon laser, Nd: YAG laser and $CO_2$ laser. (B) Photon absorption of Argon laser (488 nm and 514 nm), diode laser (940 nm) and Nd: YAG laser (1064 nm) by oxygenated or desoxygenated blood and water.

carbon (irradiation of wood or cork causes carbon to be attached to the glass fiber).

Carbon stops the wide spread of the laser beam at the tip of the fiber. In this case, the laser energy is deposited on the surface of the tissue and in the tissue just below. The effect of laser irradiation then can be observed and endoscopically controlled much better than in the non-contact mode. The penetration depth is reduced and the risk of uncontrollable side effects is very low.

For the application of laser energy we use specially designed instruments (Fig. 2) with canals

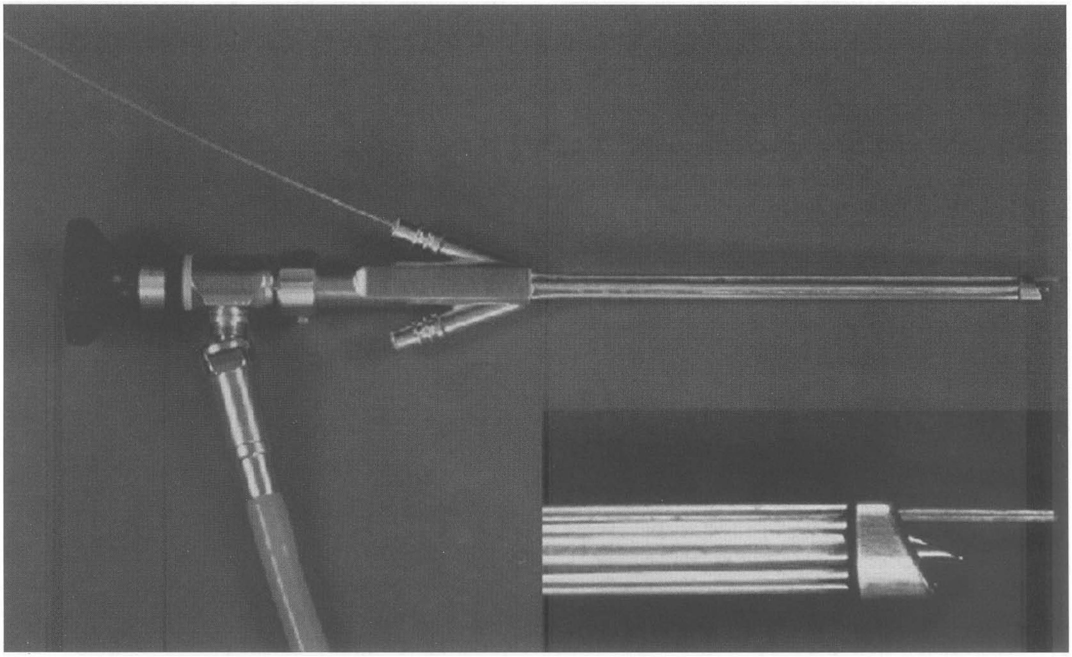

FIGURE 2 Laser application sheath according to Scherer (Richard Wolf Comp., Germany) with exchangeable telescope, laser fiber channel, and plume suction channel.

for rigid endoscopes equipped with various viewing angles, for the laser fiber and for suction. Some of the applicators have Albarran levers, with which the laser fiber can be bent (Fig. 3A,B). The laser light can be administered in a continuous way or in a pulsed mode; during the intervals the tissue can cool down reducing the degree of coagulative impact if desired.

Nasal mucosa decongestion is achieved by naphazoline containing nose drops to allow better vision. In addition, the nasal mucosa is anesthetized with a tetracaine solution. Some minutes later lidocain gel (5%) is applied onto the surgical target using cotton wool buds.

### INDICATIONS AND SURGICAL METHODS

### Hyperplasia of Nasal Turbinates

*Symptoms*: Blockage of upper airway, hypersecretion, i.e. in pollinosis or during pregnancy.

There are three turbinates in the nose, the lower one is the largest. Below each turbinate is a meatus. The size of the lower turbinate is reduced by application of a stripe-like coagulation zone at its lower margin, its coagulation diameter is around 1–2 mm. The turbinate is touched at the rear end by the laser fiber, which is then slowly pulled anteriorly (Fig. 4A–C). A white zone ("blanching") around the fiber marks the area where all vessels have been closed. As long as one does not leave this zone during movement, no bleeding will occur.

### Narrow Middle Meatus

*Symptoms*: Sinus-infections after a common cold, chronic sinusitis with mucosal swellings in the ethmoid system and maxillary sinuses.

The airway to the maxillary sinus and the ethmoid passes through the middle meatus. It is blocked by a hyperplasia of the middle turbinate, a pneumatized middle turbinate, which is thicker than normal (concha bullosa media) or a medialized lateral wall of the nose

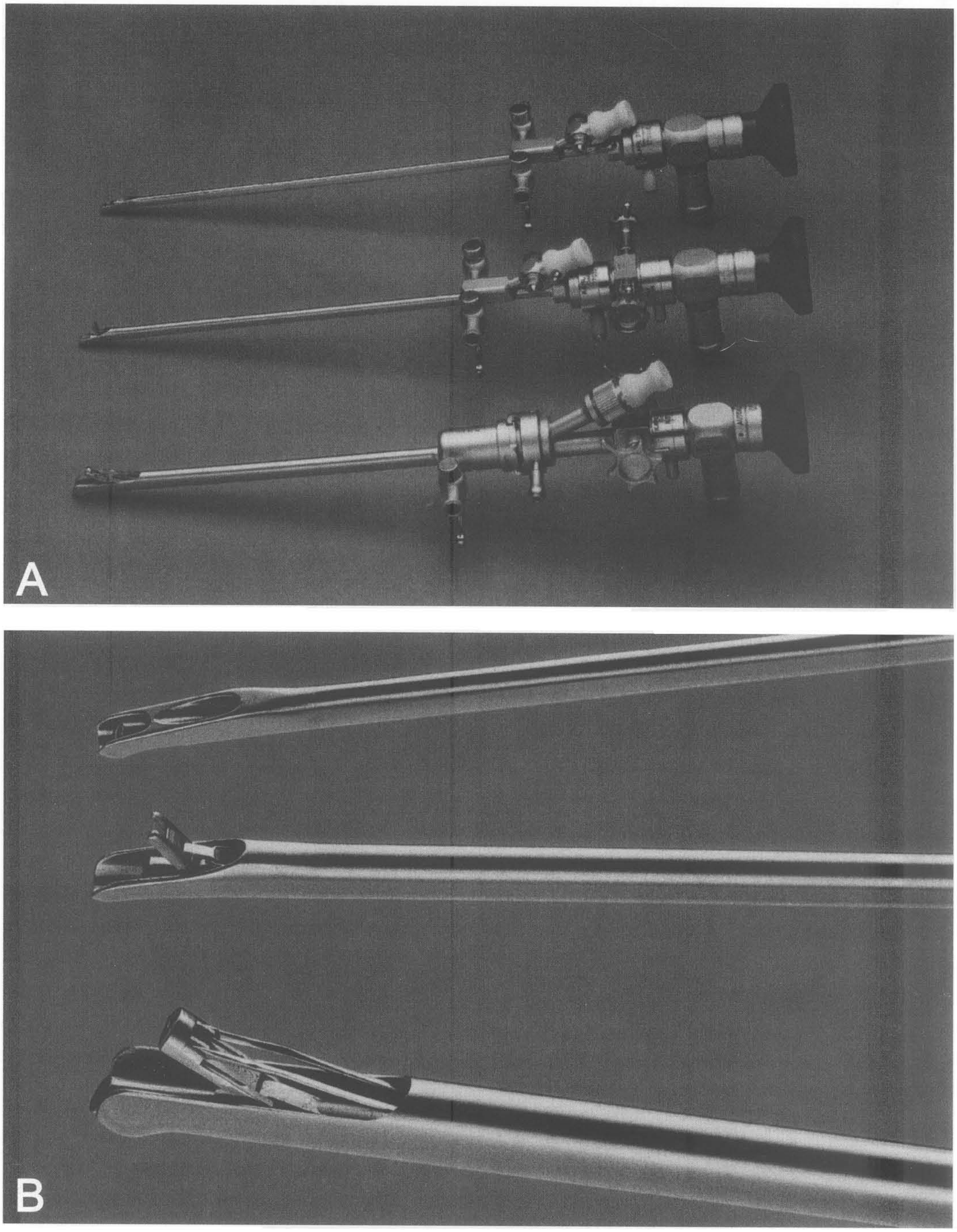

FIGURE 3 (A) Laser application sheath according to Hopf/Scherer (Karl Storz Comp., Germany) [three different outer diameters] with exchangeable side view Hopkins telescope, laser fiber channel, and plume suction channel–with or without fiber steering device at the tip. (B) Magnification of the tip of the laser application sheath according to Hopf/Scherer (Karl Storz Comp., Germany) showing the Albarran lever in two instruments and the straight forward fiber outlet in the third.

(bulla ethmoidalis, medialized uncinate process). In this case, the middle meatus is therapeutically enlarged by coagulating and vaporizing the lateral wall of the middle turbinate (Fig. 5A–F), or by opening a pneumatized middle turbinate (Fig. 6A–G). In a second laser assisted step, after primary wound healing, the ethmoid cell system is opened by resecting an ethmoid bulla or cutting off a medialized uncinate process. The sensitive ostio-meatal complex is usually left untouched. Topical corticosteroid medication is usually given after laser treatment.

### Synechia

Synechias can be found as a result of preceding sinus surgery or as a result of trauma. Usually they are

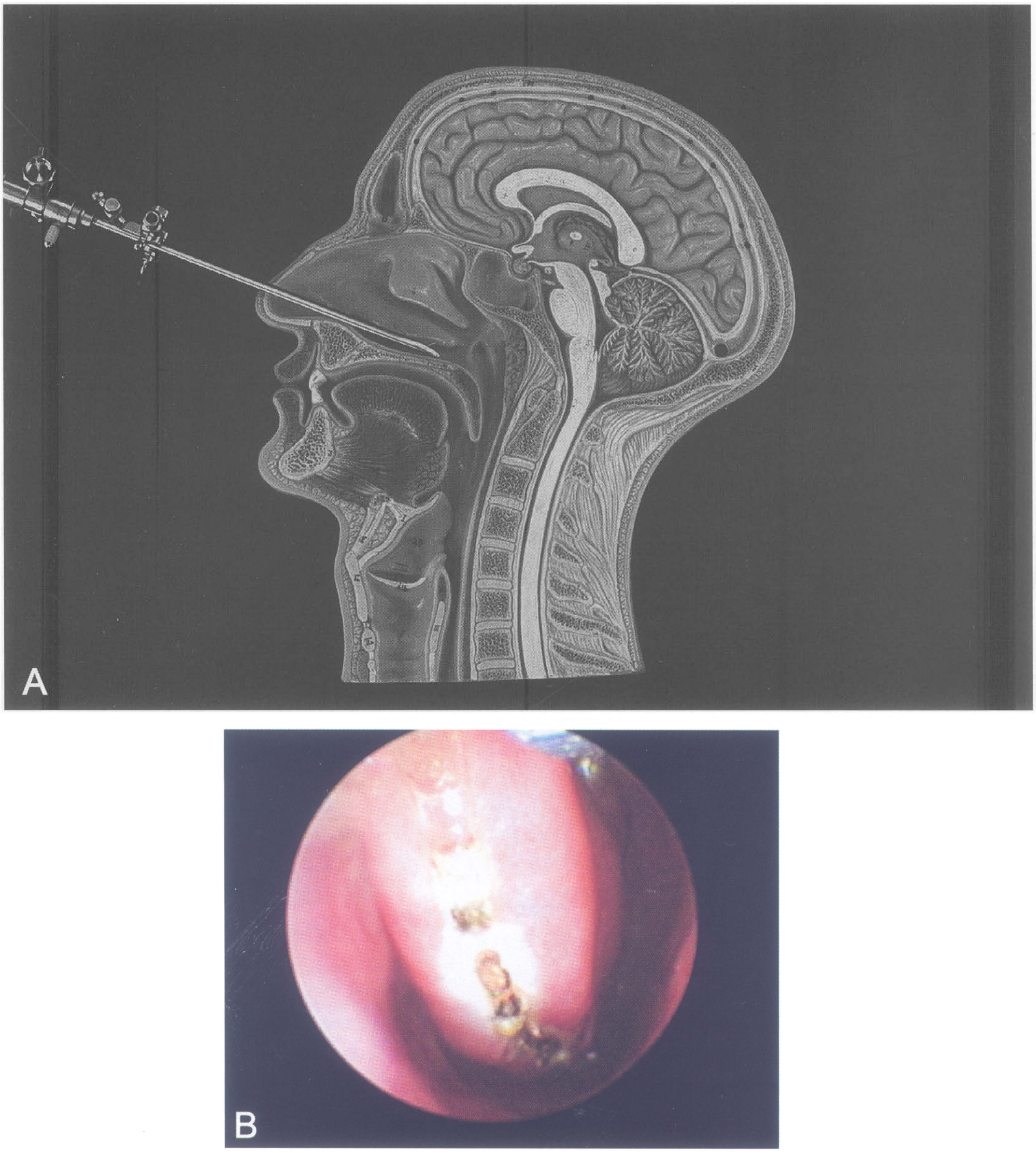

FIGURE 4 A–B.

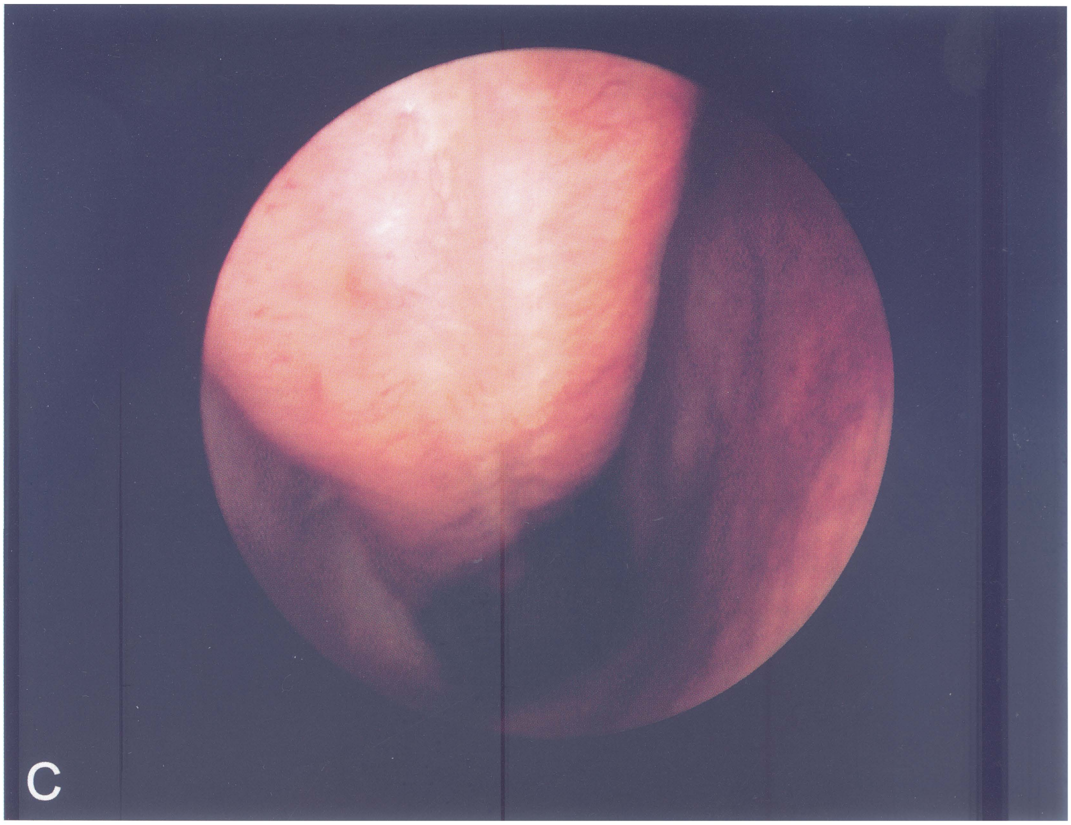

FIGURE 4 (A) Laser treatment of the right lower turbinate: Teaching model showing stripe-like coagulation starting at the rear end of the lower turbinate. (B) Laser treatment of the right lower turbinate: stripe-like bloodless coagulation at the inferior rim of the turbinate with char formation in between the blanching area. (C) Laser treatment of the right lower turbinate: surgical result without decongestion. Markedly enlarged inferior nasal meatus by good size reduction of the turbinate six weeks after diode laser surgery.

found between turbinates and the septum or between turbinates and the lateral nasal wall (lateralized middle turbinate). Provided they are not too long, they can be resected quite easily (Fig. 7).

### Septum Spurs and Ridges

Spurs and ridges are septal structures which either block or deviate the airway or irritate the nasal mucosa by direct contact with the turbinates. We cut the tip of such structures as shown in Fig. 8A. Usually the lower part of these structures contain bone. When bone is irradiated with a laser beam, and is heated up locally, it is converted into a white shining, “china like”, fragile structure (Fig. 8B), which can be easily broken by using a little force using the laser fiber. This new structure is biologically inert and is well accepted by the mucosa during re-epithelialization. Granulation tissue in this area has only occasionally been observed (Fig. 8C).

### Polyps

Polyps in the nose are due to a swelling of the mucosa, being a hyperreactive status.

They are treated locally by topical administration of corticosteroids and/or by resection. Depending on the pathogenesis of the polyps, recurrences are common.

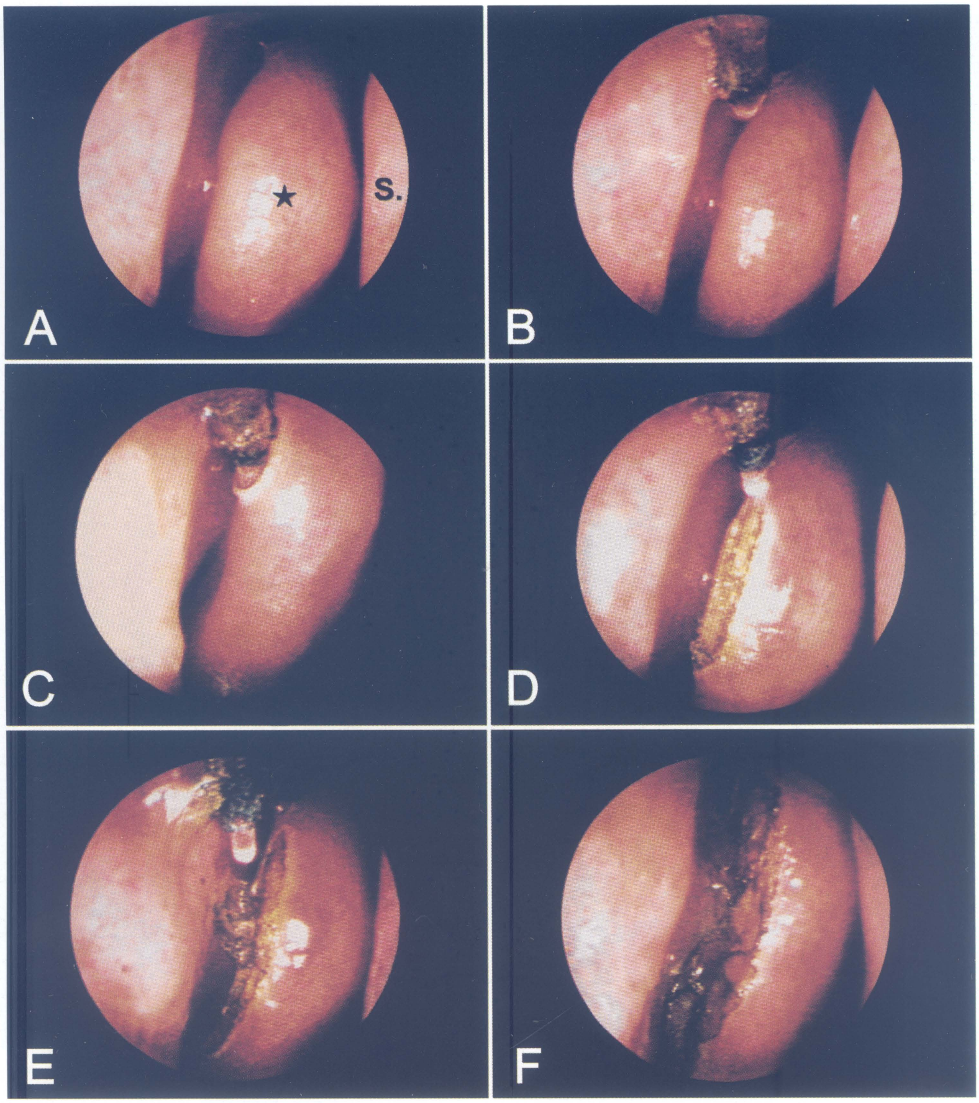

FIGURE 5 Laser assisted enlargement of the middle nasal meatus: vaporization and coagulation of the lateral wall of the middle turbinate. [★ concha media, S. septum nasi]. (A) Pre-operative situs; (B) positioning of the fiber under video-endoscopic control; (C) starting soft tissue vaporization in the pulsed mode, contact application; (D) reducing the size of the head of the middle turbinate; (E + F) continuous vaporization and coagulation of the lateral wall of the middle turbinate thus opening the access to the anterior ethmoid.

Some patients are operated on several times without relief. In the post-operative period, one can treat new developing polyps with laser coagulation as well as small primary polyps in a chronic sinusitis. This treatment is not curative but it helps the patient and gives him the ability to breathe. Polyps covering a sinusoidal ostium can be entirely removed in a curative way (Fig. 9A–D)

### Mucoceles

Mucoceles are encapsulated sinusoidal areas. Usually they are situated in the ethmoid or frontal sinus. They contain mucus and expand slowly due to ongoing mucus production. Quite often they penetrate into the orbital cavity from above or from the medial wall, causing a deviation of the eye globe.

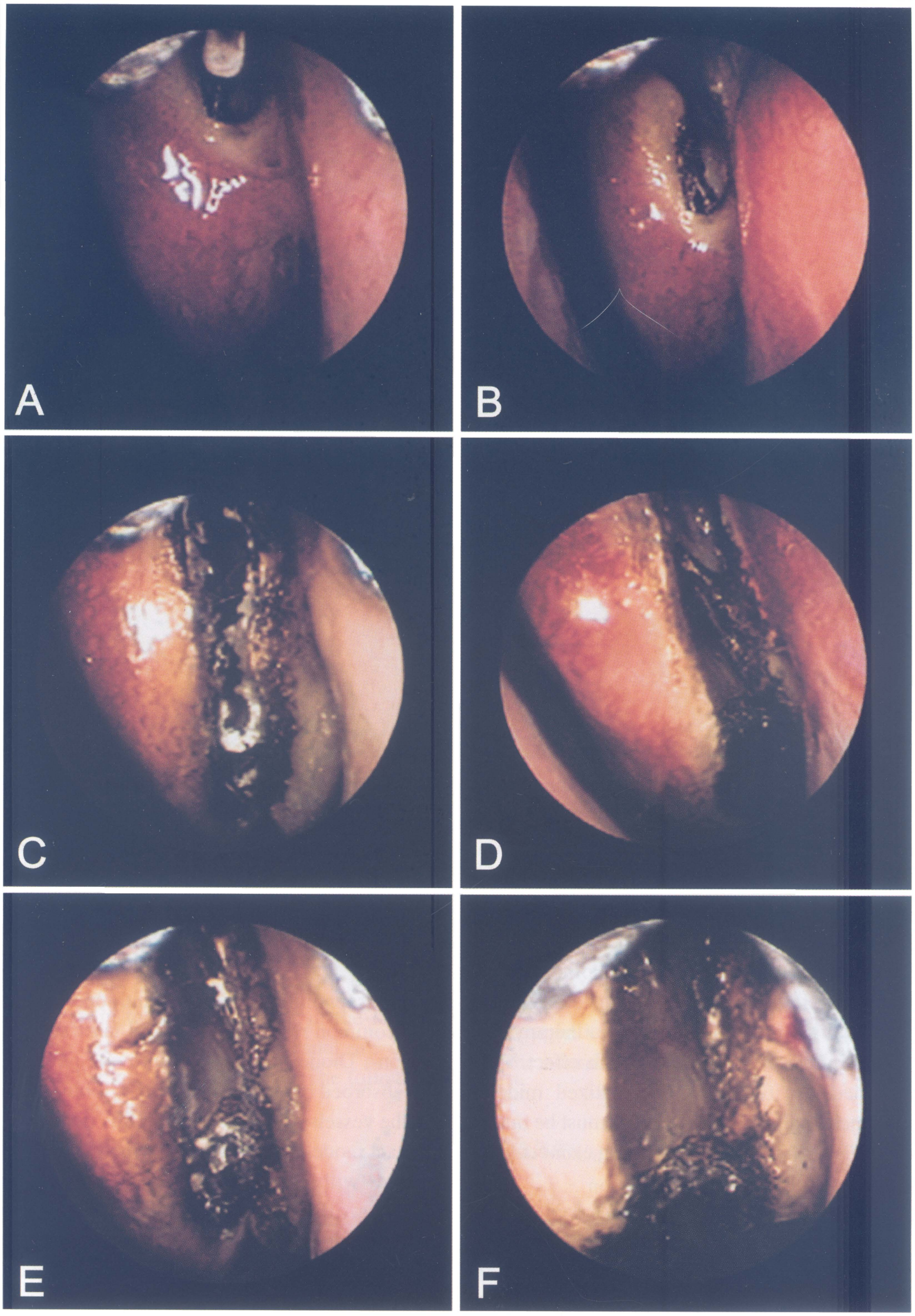

FIGURE 6 A–B.

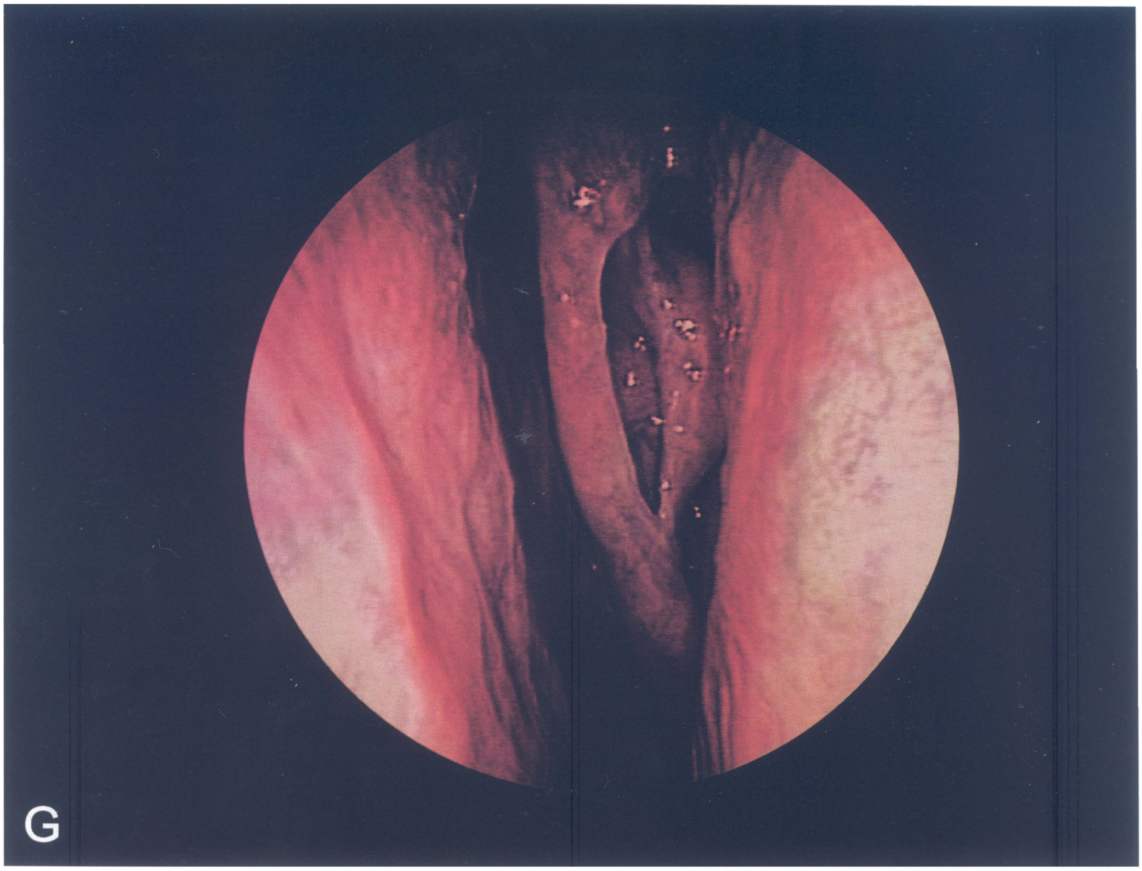

FIGURE 6 Laser assisted enlargement of the middle nasal meatus: opening and diode laser resection of the lateral wall of a concha bullosa media. (A) Positioning of the fiber under video-endoscopic control; (B) gentle surface coagulation (low power, contact application); (C) starting vertical soft and hard tissue vaporization in the pulsed mode dissecting through the bone; (D) bloodless opening of the concha bullosa is just performed; (E + F) continuous vaporization and dissection of the lateral wall of the concha bullosa creating a wide access to the anterior ethmoid; (G) routine follow up 90 days after laser surgery: wide access to the ethmoid, perfect wound healing, no scar formation.

When the anterior wall of the mucocele is visible endoscopically, laser treatment is the therapy of choice with minimal effort and maximum effect. The wall is opened, as it is in a pneumatized middle turbinate (concha bullosa). The opening must be made big enough (Fig. 10A–E) so that scarring cannot close it up again (Fig. 10F–G)

### Epistaxis and Osler's Disease

Conventional surgical treatment of epistaxis, especially in Osler's disease, is challenging and often disappointing because of intraoperative bleeding and common recurrence. Special laser such as KTP-, Argon-, Diode 940 nm laser provide photons, which are absorbed by oxygenated hemoglobin and thus heat up the vessels specifically. With this treatment in non-contact mode, most of the participating arteries can be closed without the occurrence of intraoperative bleeding. Bigger vessels in the anterior area of the nose are protected and temporarily compressed by a cooled glass spatula through which the laser beam is transmitted (Fig. 11A–D) ("Compression-coagulation-method").

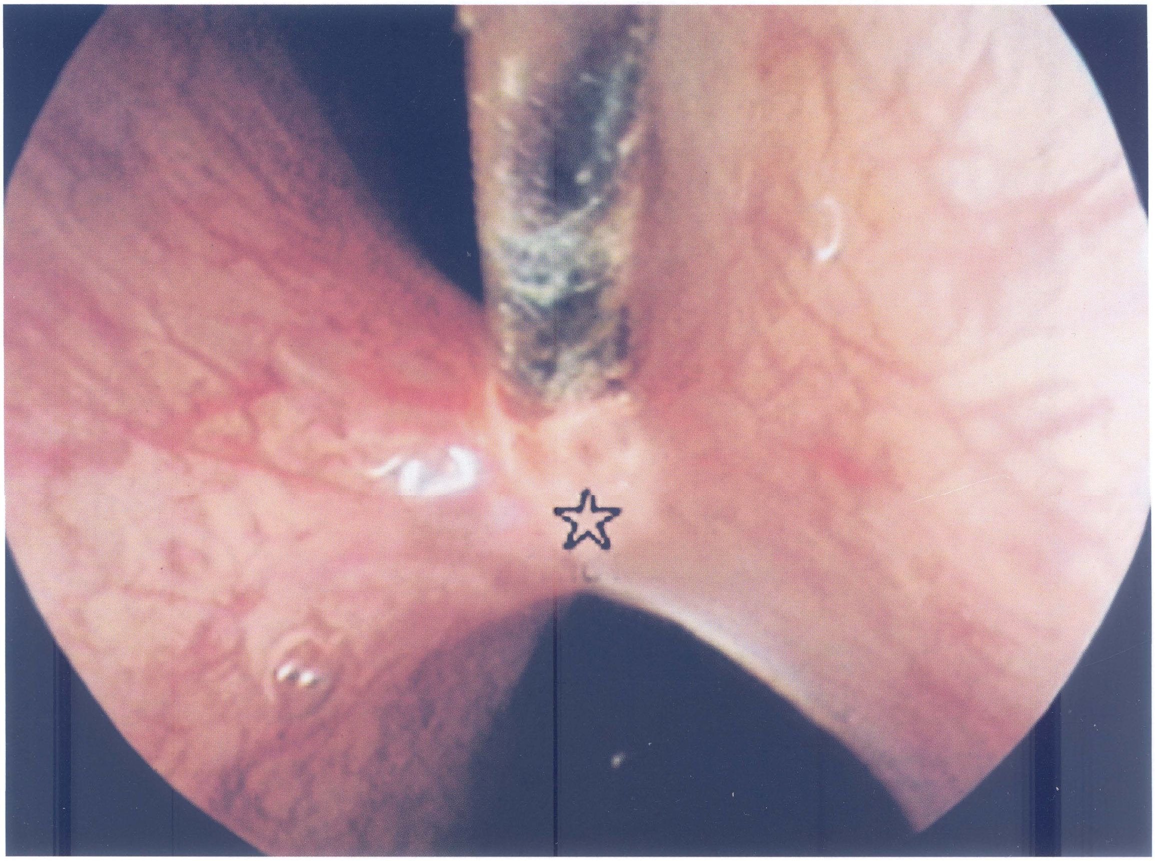

FIGURE 7 Laser assisted resection of septo-turbinal synechia[☆]: vaporization of the synechia and subsequent coagulation of the roots of the soft tissue bridge in contact mode.

### Transnasal Laser Surgery

For endonasal laser surgery, interventions in the nasopharynx and at the rear side of the soft palate, rigid endoscopes are used quite exclusively. For some indications deep in the oropharynx or in the larynx, flexible endoscopes with a working canal are used, through which the laser fiber is guided. The following diseases are treated up to now

*with rigid endoscopes*

- adenoids in adults,
- chronic swelling of the pharyngeal opening of the Eustachian tube
- cysts of the nasopharynx
- papillomas of the backside of the soft palate

*with flexible endoscopes*

- chronic edema of the supraglottic region after irradiation
- cysts of the vallecula or of the false vocal cord
- lymphatic plaques at the base of the tongue or at the lateral pharyngeal wall (small areas)

### RESULTS

The effect of laser treatment was analyzed in 44 patients with rhinomano-, rhinoresisto- and acoustic rhinometric measurements. Thirty of them were treated with a coagulation of the lower turbinate only (group 1), 14 of them in combination with an

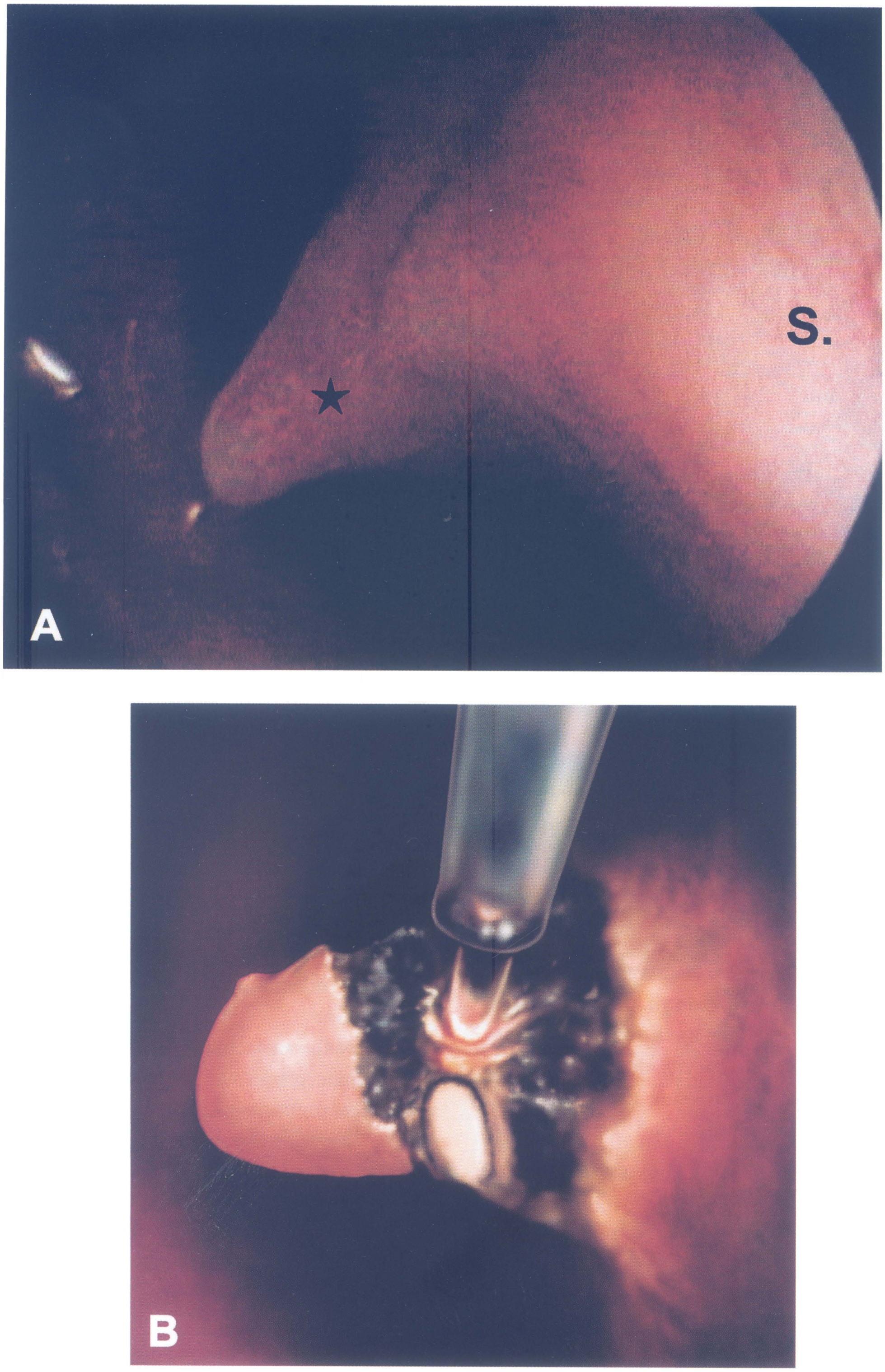

FIGURE 8 A–B.

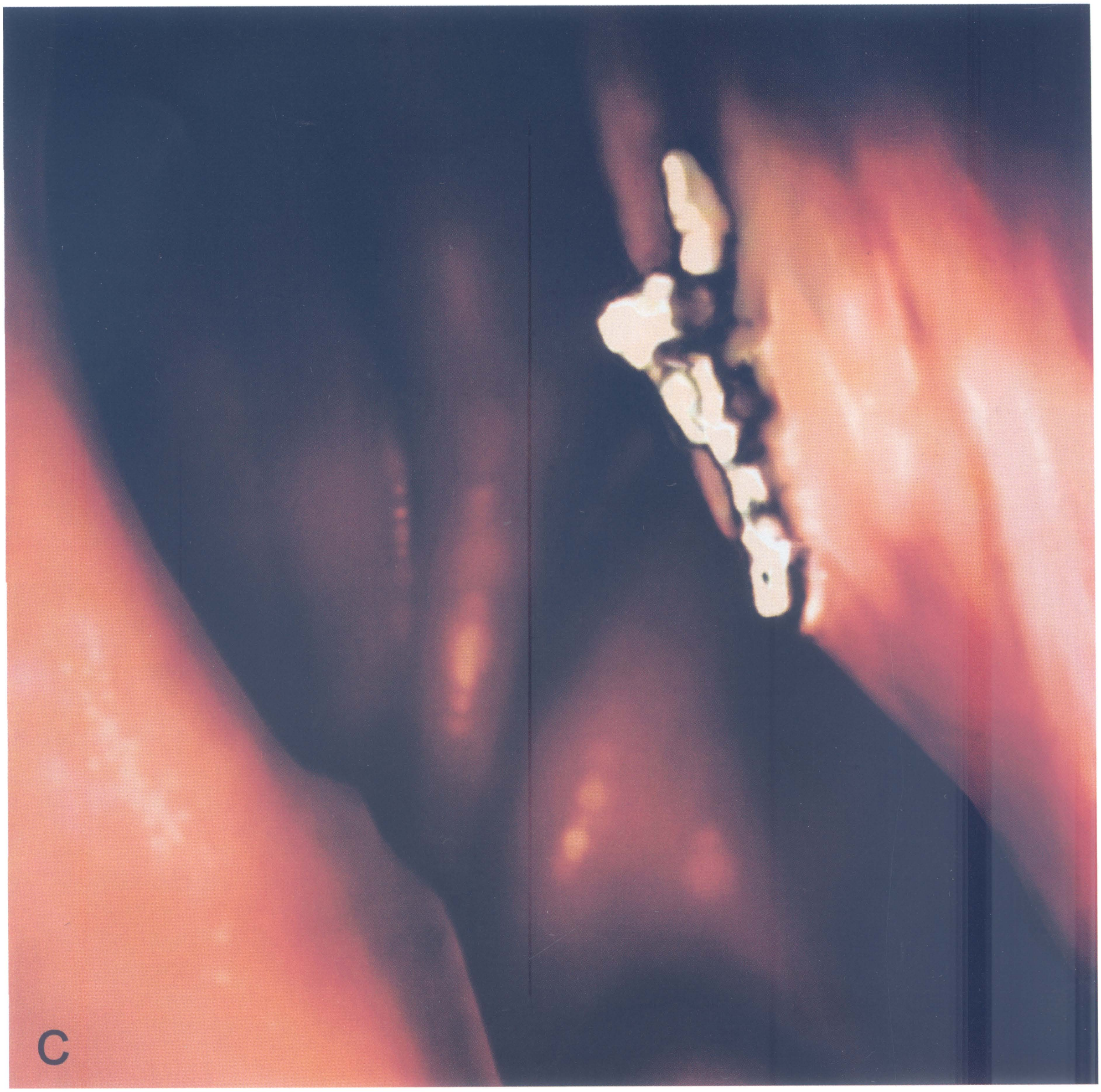

FIGURE 8 Laser assisted resection of a septal spur: (A) sharp extending bony spur [★] of septum nasi [S.], pre-operative situation; (B) after gentle superficial mucosal coagulation (low power, continuous mode) the vaporization in pulsed mode (high laser power, short exposure time, long intervals) is performed. Porcelain-like transformation of the bone during treatment; (C) situation one week after laser resection, complete re-epithelialization will happen within 3–6 weeks.

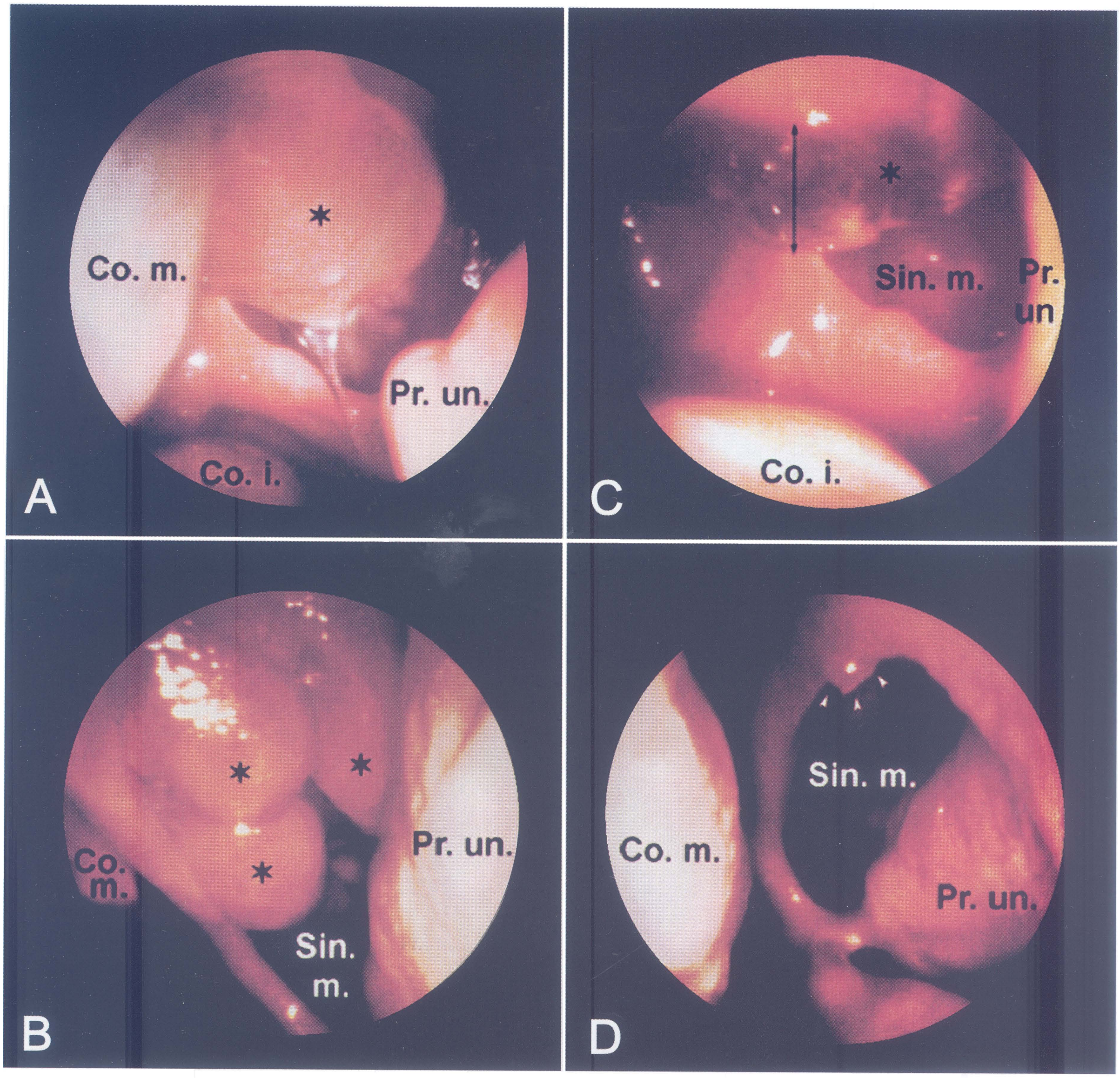

FIGURE 9 Laser treatment of a chronic polypous rhinosinusitis recurrency: (Polyp [∗], Concha media [Co. m.], Concha inferior [Co. i.], Processus uncinatus [Pr. un.], Sinus maxillaris [Sin. m.]). (A + B) maxillary sinus ostium subtotally blocked by a polypous soft tissue (one star and three stars, pictures left row); (C) Situation after polyp coagulation(low power, contact application); (D) 6 weeks after minimal invasive laser treatment: the ostium is wide open again.

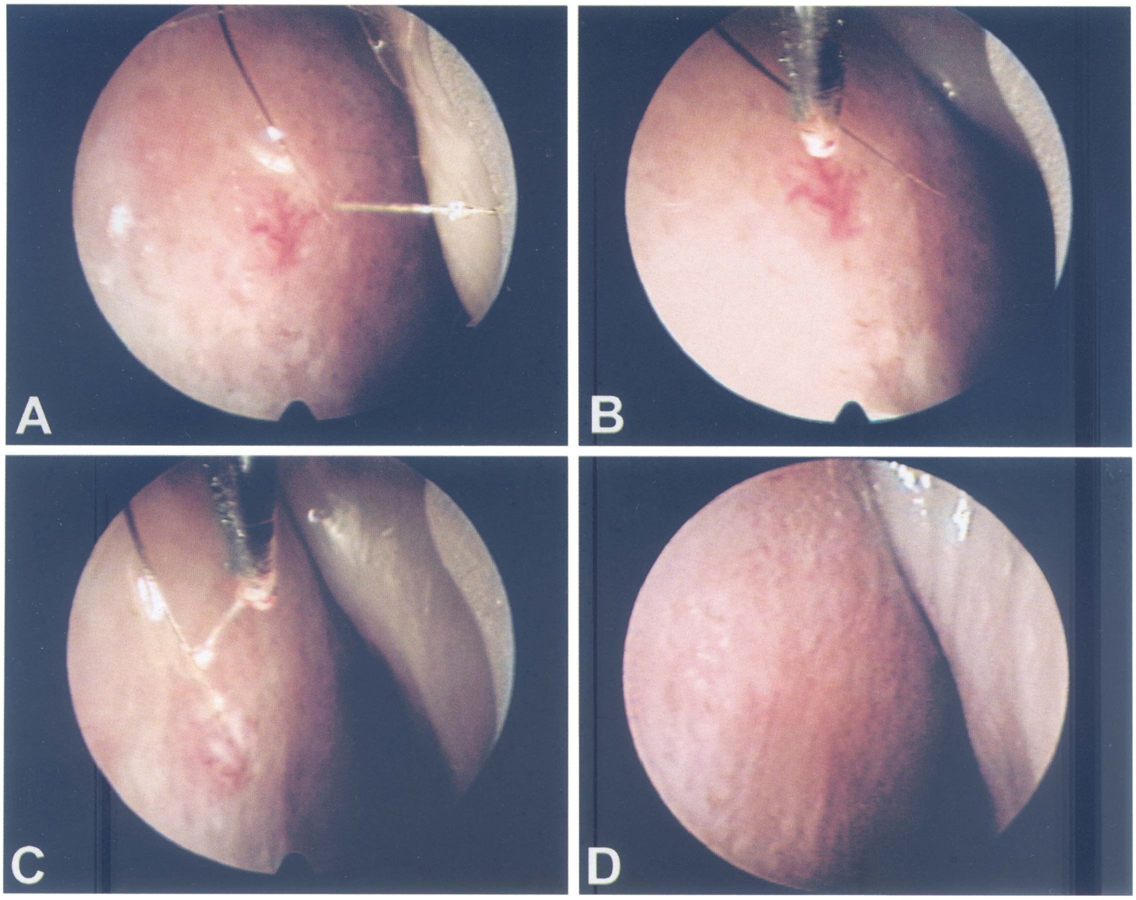

FIGURE 11 Laser treatment of a septal telangiectasia causing recurrent epistaxis. (A) pre-operative situation at the left side of the septum; (B) positioning of the fiber, non-contact mode; (C) situation immediately after treatment; (D) perfect wound healing, routine follow up 6 weeks after laser surgery.

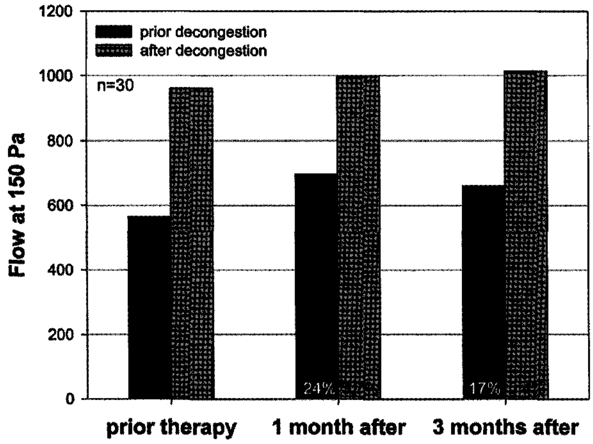

FIGURE 12 Rhinomanometric measurement of nasal air flow in 30 patients of group 1 (laser coagulation of the lower turbinate). The percentages indicate the improvement after therapy.

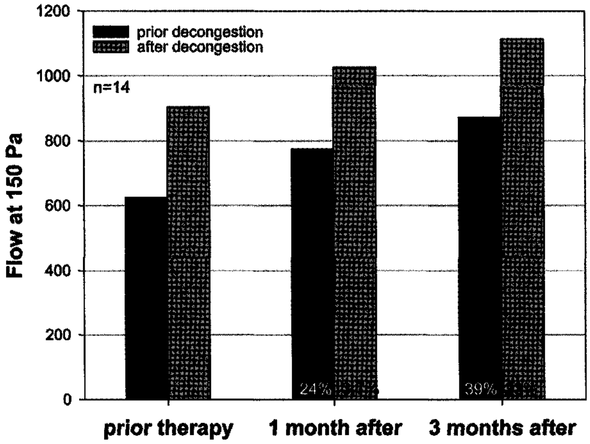

FIGURE 13 Rhinomanometric measurement of nasal air flow in 14 patients of group 2 (laser coagulation of the lower turbinate and laser assisted enlargement of middle meatus). The percentages indicate the improvement after therapy.

TABLE I Subjective success rate

| | Group 1 | | | Group 2 | | |
|---|---|---|---|---|---|---|
| | 3 Months (%) | 9 Months (%) | 12–15 Months (%) | 3 Months (%) | 9 Months (%) | 12–15 Months (%) |
| Not satisfied (%) | 13 | 18 | 18 | 0 | 21 | 14 |
| Satisfied (%) | 47 | 29 | 47 | 50 | 36 | 57 |
| Very satisfied (%) | 40 | 53 | 35 | 50 | 42 | 29 |

enlargement of the middle meatus (group 2). The success rate concerning nasal air flow one and three months after treatment is shown in Figs. 12 and 13. It is higher and more permanent in patients, in whom, both the lower and the middle meatus were enlarged. The percentages shown in Figs. 12 and 13 indicate the improvement after therapy.

Patients were asked to tell us their subjective success classification after 3, 9 or 12–15 months (Table I). About 80% of the patients in both groups were either satisfied or very satisfied, most of them very satisfied in the first year. This rate tends to reverse in both groups in the time thereafter.

In addition to the measurement we asked the patients to fill out a questionnaire on intra- and postoperative pain (Tables II and III). Intra-operative pain was moderate, low or absent in 90% of the patients and post-operative in 92%. Patients suffering from a lot of pain reported that they had the same trouble when treated by dentists. In these patients, we now prolong the pre-operative anesthetic packing to 40 min and renew it several times. In rare cases we inject 2% tetracain with adrenaline 1:200 000 to surgical target. In severe cases, midazolam sedation is titrated intravenously.

Another group of patients suffer from a hyper-reactivity of their nasal mucosa. They tend to have pain during laser-treatment. In these patients superficial anesthesia does not work due to their hypersecretions, which washes out the anesthetic drug very quickly. An oral anti-histaminic pre-medication for one week and a prolonged package reduces the intra-operative pain effectively.

The subjective ratio for postoperative bleeding is shown in Table IV. The rate of absent or low bleeding (traces of blood in the handkerchief) is 73% in group 1 and 80% in group 2. In two cases of the described groups (group 1) we had to stop a small bleeding from the coagulation zone of the lower turbinate in the immediate postoperative period by applying a

TABLE II Intra-operative pain

| Group 1 | | | | Group 2 | | | |
|---|---|---|---|---|---|---|---|
| Strong | Moderate | Low | Absent | Strong | Moderate | Low | Absent |
| 3 | 7 | 5 | 15 | 3 | 1 | 4 | 6 |
| 10% | 23% | 17% | 50% | 21% | 7% | 28% | 42% |

TABLE III Post-operative pain

| Group 1 | | | | Group 2 | | | |
|---|---|---|---|---|---|---|---|
| Strong | Moderate | Low | Absent | Strong | Moderate | Low | Absent |
| 1 | 8 | 2 | 19 | 0 | 2 | 4 | 8 |
| 3% | 27% | 7% | 63% | 0% | 14% | 29% | 57% |

TABLE IV Post-operative bleeding

| Group 1 | | | | Group 2 | | | |
|---|---|---|---|---|---|---|---|
| Strong | Moderate | Low | Absent | Strong | Moderate | Low | Absent |
| 2 | 6 | 8 | 14 | 1 | 4 | 3 | 7 |
| 7% | 20% | 26% | 47% | 7% | 29% | 21% | 50% |

decongestive package to the nose for a short lasting period.

### DISCUSSION

The endoscopically based endo- and transnasal laser surgery offers a therapeutic strategy, which achieves a maximum effect with minimum effort. Rhinomanometric measurements (Figs. 12 and 13) support this statement. Superficial anesthesia of the mucosa is sufficient in most cases and pain during and after the procedure is absent, low or moderate in a high percentage. There is no or minimal bleeding during the tissue coagulation. In the post-operative period patients usually have more or less bloody secretions for several days.

Infections in the operated area are extremely rare and so post-operative care is rendered unnecessary or is limited to one or two follow-up appointments. This kind of minimal invasive therapy is well-accepted by the patients.

The subjective success classification of the patients (Table I) and the measurement in patients of group 1 show a decline in the therapeutic effect after several months. This happens predominantly in young persons, in who the cavernous tissue tends to expand again. A second laser session in this group usually leads to a permanent success.

The method described here has been used in our clinic since 1987. We started with the coagulation of hyperplastic turbinates and have added other indications step by step. Up to now, more than 5500 patients have been treated. We have installed a special room in our outpatient department for this kind of surgery, equipped with three different coagulative lasers (Diode laser 940 nm, medilas D fibertom SkinPulse S, Dornier Medizinlaser GmbH [Germany]; Nd:YAG laser 1064 nm, MY 60, Martin Medizintechnik [Germany]; Diode laser 810 nm, 60 20, Lumenis-ESC-Sharplan). Each of these lasers is suitable for this treatment.

In most cases, we use the diode laser 940 nm. Its laser–tissue interaction is optimal and the different parameters, it offers, are suitable for all our needs and procedures. Other lasers with shorter wavelengths (KTP and Argon) can also be used. Their photons are highly absorbed in hemoglobin and, therefore, they are effective for a reduced coagulation zone. The intraoperative control of tissue coagulation is highly effective, so that the procedure can be performed at an early stage of medical doctors' training.

In rare cases, we have had complications and side effects. We rarely observed some synechias (8 patients) when we created wounds on opposing sides and thick fibrinous tissue gave rise to the formation of a tissue bridge. We have also experienced intra-operative bleeding in a few cases when the laser fiber has been moved too rapidly in the tissue before all surrounding vessels had been closed (blanching around the fiber). The bleeding could be stopped by decongestive drugs on cotton wool buds. Only one patient had been hospitalized and surgically treated for a post-surgical bleeding 8 days after endonasal laser surgery. No perforations of the septum have been observed up to now.

### *References*

[1] Messerklinger, W. (1966) "Über die Drainage der menschlichen Nasennebenhöhlen unter normalen und pathologischen bedingungen. 1", *Mitteilung. Mschr. Ohrenheilk.* **100**, 56–68.

[2] Messerklinger, W. (1979) "Das Infundibulum ethmoidale und seine entzündlichen Erkrankungen", *Arch. Otolaryngol.* **222**, 11–22.

[3] Messerklinger, W. (1987) "Die Rolle der lateralen Nasenwand in der Pathogenese, Diagnose und Therapie der rezidivierenden und chronischen Rhinosinusitis", *Laryngol. Rhinol. Otol.* **66**, 293–299.

[4] Wigand, M.E. (1989) Endoskopische Chirurgie der Nasennebenhöhlen und der vorderen Schädelbasis (Thieme-Verlag, Stuttgart).

[5] Wigand, M.E., Steiner, W. and Jaumann, M.P. (1978) "Endonasal sinus surgery with endoscopical control: from radical operation to rehabilitation of the mucosa", *Endoscopy* **10**, 255–260.

[6] Stammberger, H. (1985) "Unsere endoskopische Operationstechnik der lateralen Nasenwand—ein endoskopisch-chirurgisches Konzept zur Behandlung entzündlicher Nasennebenhöhlenerkrankungen", *Laryngol. Rhinol. Otol.* **64**, 559–566.

[7] Kennedy, D. (1985) "Functional endoscopic sinus surgery", *Arch. Otolaryngol.* **111**, 643–649.

[8] Rudert, H. (1988) "Mikroskop- und endoskopgestützte Chirurgie der entzündlichen Nasennebenhöhlenerkrankungen", *HNO* **36**, 475–482.

[9] Cook, J.A., Mc Combe, A.W. and Jones, A.S. (1993) "Laser treatment of rhinitis—one year follow-up", *Clin. Otolaryngol.* **18**, 209–211.

[10] Dobrovic, M. and Hosch, H. (1994) "Non-contact applications of Nd: YAG laser in nasal surgery", *Rhinology* **32**, 71–73.

[11] Elwany, S. and Harrison, R. (1990) "Inferior turbinectomy: comparison of four techniques", *J. Laryngol. Otol.* **194**, 206–209.

[12] Feyh, J. (1995) "Endoscopic surgery of the nose and paranasal sinuses with the aid of the holmium: YAG laser". In: Rudert, H., Werner, J.A. (Hrsg), Lasers in Otorhinolaryngology and in Head and Neck Surgery, *Adv. Otolaryngol.*, (Basel, Karger), **49**, 122–124.

[13] Fukutake, T. (1993) "*$CO_2$ laser and turbinate dysfunktion*". Presented at *The XII International Symposium on Infection and Allergy of the Nose* (ISIAN), Seoul, Korea, October 8–11, 1993.

[14] Hopf, J.U.G., Hopf, M., Gundlach, P. and Scherer, H. (1998) "Miniature endoscopes in oto-rhino-laryngologic applications", *Min. Invas. Ther. Allied Technol.* **7/3**, 209–218.

[15] Hopf, J.U.G., Hopf, M. and Koffroth-Becker, C. (1999) "Minimal invasive Chirurgie obstruktiver Erkrankungen der Nase mit dem Diodenlaser", *Laser Med.* **14/4**, 106–115.

[16] Hopf, J.U.G., Hopf, M. (2001) In: Scherer, H., ed, FEELS—Functional Endoscopic Endonasal Laser Surgery (Endo-Press, Tuttlingen).

[17] Jovanovic, S. and Dokic, D. (1995) "Nd: YAG-Laserchirurgie in der Behandlung der allergischen Rhinitis", *Laryngol. Rhino. Otol.* **74**, 419–422.

[18] Kamami, Y-V. (1997) "Laser-assisted outpatient septoplasty results on 120 patients", *J. Clin. Las. Med. and Surg.* **15**, 123–129.

[19] Scherer, H., Reichert, K. and Schildhauer, S. (1999) "Die Laserchirurgie des mittleren Nasenganges bei der rezidivierenden Sinusitis", *Laryng. Rhino. Otol.* **78**, 50–53.

[20] Shapshay, S.M., Rebeiz, E.E., Michail, M. and Pankratov, M.M. (1992) "Holmium: yttrium aluminium garnet laser-assisted endoscopic sinus surgery: clinical experience", *Laryngoscope* **101**, 142–149, 18.

[21] Ohyama, A., Yamashita, K., Furuta, S., Nobori, T. and Daikuzono, N. (1988) "Applications of the Nd: YAG laser in otorhinolaryngology", In: Joffe, S.N. and Oguro, Y., eds, Advances in Nd: YAG Laser Surgery (Springer, Berlin, Heidelberg, New York), pp. 156–178.

[22] Ohyama, M. (1989) "Laser Polypectomy", *Rhinology* **8**(Suppl.), 35–43.

[23] Krespi, Y.P. and Slatkine, M. (1994) "Nd: YAG fiber delivery system for submucosal interstitial coagulation of nasal turbinates", *Laser Surg. Med.* **15**, 217–248.

[24] Lenz, H. (1985) "Acht Jahre Laserchirurgie an den unteren Nasenmuscheln bei Rhinopathia vasomotorica in Form der laserstrichkoagulation", *HNO* **33**, 422–425.

[25] Levine, H.L. (1989/1) "Lasers and endoscopic rhinologic surgery", *Otolaryng. Clin. N. Am.* **22**(4), 739–748.

[26] Lippert, B.M. and Werner, J.A. (1995) "Reduction of hyperplastic turbinates with the $CO_2$-laser". In: Rudert, H., Werner, J.A. (Hrsg), Lasers in Otorhinolaryngology, and in Head and Neck Surgery. *Adv. Otorhinolaryngol.*, (Basel, Karger), **49**, 118–121.

[27] Mehta, A.C., Livingston, D.R. and Levine, H.L. (1987) "Fiberoptic bronchoscope and Nd: YAG laser treatment of severe epistaxis from nasal hereditary hemorrhagic telangiectasia and hemangioma", *Chest* **91**, 791–792.

